# Protein Citrullination in Amyotrophic Lateral Sclerosis and Other Neurodegenerative Diseases

**DOI:** 10.33696/neurol.5.101

**Published:** 2024

**Authors:** Issa O. Yusuf, Webb Camille, Paul R. Thompson, Zuoshang Xu

**Affiliations:** 1 Department of Biochemistry and Molecular Biotechnology, University of Massachusetts Chan Medical School, Worcester, MA 01605, USA; 2 Program in Chemical Biology, University of Massachusetts Chan Medical School, Worcester, MA 01605 USA

**Keywords:** Alzheimer’s disease, Neurodegenerative disease, Parkinson’s disease

## Abstract

Protein citrullination (PC) is a posttranslational modification (PTM) that converts a peptidyl arginine into a peptidyl citrulline. Aberrant PC is a hallmark of neurodegenerative diseases, including amyotrophic lateral sclerosis (ALS), Alzheimer’s disease, Parkinson’s disease, prion disease, and multiple sclerosis. Common among these diseases is a dramatic increase of PC in reactive astrocytes. Some citrullinated proteins have been identified. The most prominent are astrocytic cytoskeletal proteins such as GFAP and vimentin, and myelin protein MBP. Recent investigation in ALS has revealed new changes, including a decreased PC in neurons and an association of PC with myelin protein aggregates. These findings suggest that PC contributes to protein aggregation, neuronal dysfunction, neuroinflammation, and axonal degeneration. However, how PC impact neurodegeneration remains to be understood. Further studies are needed to understand a range of questions, from how PC modulates individual protein functions to its impact on diseases. Because of the PC’s robust changes in neurodegenerative diseases, there are also prospects that this PTM may be harnessed as biomarkers, and modulation of this PTM may be an avenue for therapy. In this review, we summarize the current understanding of PC in ALS and other neurodegenerative diseases, the investigative methods for PC, and PC’s potential as a biomarker and a therapeutic target.

## Introduction

Proteins are critical cellular machines that control cell growth, replication, and biological function. However, these proteins are encoded by a limited number of genes in our genome. To increase both the structural and functional diversity of the proteome to meet the demands in various environments, individual proteins can be posttranslationally modified in specific regions after synthesis. Several hundred posttranslational modifications (PTMs) are currently known, including phosphorylation, methylation, acetylation, glycosylation, ubiquitination, and SUMOylation [1]. Compared to these well-known PTMs, protein citrullination (PC) is relatively less studied, but has emerged to be a critical player in innate immunity. A well-established example is the formation of neutrophil extracellular traps (NETs), which trap pathogens (bacteria, viruses and parasites) for destruction during infections. Citrullination of histones decondenses chromatin and releases DNA strands to form NETs [2]. PC converts arginine residues in proteins (peptidyl-arginine) into citrullines (peptidyl-citrulline). Since the side chain of arginine is positively charged at physiological pH, this hydrolysis reaction results in loss of a positive charge, thereby altering the protein’s overall charge distribution. This PTM also alters the hydrogen bonding characteristics of the modified arginine, leading to protein structural and functional changes, including its activity and interactions with other proteins and nucleic acids [3-5]. In the case of NETs, PC weakens interaction between histones and DNA, thereby facilitating the unraveling of chromatin and DNA release.

PC is catalyzed by a family of enzymes referred to as the protein arginine deiminases (PADs). Mammals express five PADs, namely PAD1-4 (active), and PAD6 (inactive) [5]. All PAD isozymes show extensive sequence homology conserved across species. They are, however, differentially expressed in tissues, with PAD2 being the most ubiquitously expressed, and most dominant in the central nervous system (CNS) [5,6]. PAD activity is tightly regulated by calcium levels. Under normal cellular calcium concentrations (~100 nM), the PADs do not bind calcium and adopt a conformation that is catalytically inactive. Upon pathological or pharmacological stimulation, the intracellular concentrations of calcium rise, and the PADs bind to four to six calcium ions depending on the specific PAD. Calcium binding triggers a series of conformational changes that form and stabilize the active site enabling catalysis [7]. Once activated, PADs citrullinate a wide range of protein substrates, and this modification regulates several cellular processes, including cell signaling, immune responses, and gene regulation [5,8]. PADs and citrullination are involved in many inflammatory pathologies, including atherosclerosis, rheumatoid arthritis, COVID-19, lupus, cancers, and neurodegenerative diseases [3,9,10]. In neurodegenerative diseases, aberrant PAD2 activity and protein citrullination have been shown in Alzheimer’s disease (AD), Parkinson’s disease (PD), prion diseases, multiple sclerosis (MS), ischemic and traumatic brain injuries [11-16], and recently, in ALS [17,18]. In this review, we will summarize the current understanding of PC in ALS and other neurodegenerative diseases and its potential as both a biomarker and therapeutic target.

## Protein Citrullination Changes are a Common Pathological Hallmark of Neurodegenerative Diseases

Neurodegenerative diseases are a diverse group of disorders of the nervous system that are caused by a progressive loss of one or multiple groups of neurons leading to manifestation of specific clinical symptoms that are associated with the loss of neurons. These diseases increasingly affect people globally as the population ages. Pathologically, these diseases share common characteristics such as unique genetic mutations as well as sporadic cases, relentless protein aggregation, aberrant RNA processing, cytoskeletal disruption, oxidative stress, altered energy metabolism, neuroinflammation, and neuronal cell death [19]. Additionally, significant changes in PTMs and their contribution to pathogenesis are widely recognized [1]. Compared with other PTM changes, PC and PAD dysregulation are relatively understudied, but are increasingly recognized as hallmarks of these diseases (Table 1).

ALS is a deadly neurodegenerative disease that causes progressive motor neuron loss, motor dysfunction, and paralysis [20]. The role for PC in ALS has only recently been appreciated. In 2018, Tanikawa and colleagues showed that a group of RNA binding proteins that are known to be mutated in ALS, i.e., FUS, EWS, TAF15, and hnRNPA1, are citrullinated in PAD4-overexpressing cancer cells [21]. Notably, mutations in these proteins are known to enhance their propensity to aggregate but citrullination inhibited their aggregation. Furthermore, a polymorphism linked to reduced PAD4 expression appeared to increase the risk of ALS. This study suggests that a reduction in PAD4 activity could promote the onset of ALS [21]. However, these observations remain to be confirmed in ALS animal models and patients.

More recently, alterations in PC were found in both human postmortem tissues and two mouse models of ALS that express ALS-causing mutants SOD1^G93A^ and PFN1^C71G^, respectively. In both models, PC was greatly increased in astrocytes and this increase correlates spatially with regions of active neurodegeneration, and temporally with disease progression. In the spinal cord white matter, PC is present prominently in protein aggregates that colocalize with the myelin proteins PLP and MBP. Interestingly, the increase in PC is not uniform in all cells. In neurons, PC is decreased at disease onset, and this decrease persists during disease progression [18]. These findings were validated in a follow-up study on human sporadic ALS (sALS) [17]. These results suggest that changes in PC play a role in ALS disease progression, possibly by contributing to neuronal dysfunction, protein aggregation, and neuroinflammation.

AD is the most common aging-associated neurodegenerative disease characterized by loss of neurons in the cortex and hippocampus, and accumulation of senile plaques and neurofibrillary tangles in the affected brain areas. Clinically, AD manifests as progressive loss of memory and dementia [22,23]. Ishigami and colleagues examined PC in AD and showed increased PAD2 expression and PC in reactive astrocytes in the hippocampus of AD brains [24]. Since then, several studies have further characterized aberrant citrullination in AD brains. Nicholas showed that citrullinated proteins are enriched in the nucleus of neurons with cytoplasmic phosphorylated tau and in reactive astrocytes surrounding extracellular amyloid plaque in the frontal cortex [25]. Acharya and colleagues reported that PAD4 and citrullinated proteins accumulate in the cytoplasm of cortical pyramidal and hippocampal hilar neurons expressing Aβ42 [26]. Mukerjee *et al.,* showed that Aβ is citrullinated in sporadic and familial AD brains at arginine 5 [27]. Increased citrullination was also observed in the brains of AD with cerebrovascular disease [28]. The significance of the increased PC is yet to be established but likely contributes to neuroinflammation [28].

PD is a chronic, progressive neurodegenerative disease characterized by loss of dopaminergic neurons in the substantia nigra pars compacta, neuronal α-synuclein inclusions, and the resulting motor symptoms including bradykinesia, static tremor, and motor rigidity. PD is also associated with cognitive impairments [29-31]. In the PD brain, citrullinated proteins were detected in astrocytes in the substantia nigra (SN), but appeared more prominent in surviving dopamine neurons, where citrullinated proteins are diffusely distributed in the cytoplasm predominantly in the cell bodies and occasionally in proximal dendrites [16]. In the prefrontal cortex of X-linked Dystonia Parkinsonism, PAD2 and PAD4 are increased together with histone H3 citrullination [32]. A significant increase in PC and PAD expression levels was also observed in the anterior cingulate cortex and hippocampus in the earlier stages of PD [33]. In a rat model of pre-motor PD, PC was increased in the vasculature of cortex and hippocampus. Furthermore, a significantly high number of proteins were citrullinated in the plasma and plasma extracellular vesicles [34]. The early detection of PC in different models of PD suggests that aberrant citrullination is involved in the pathogenesis of this disease.

Prion diseases are a group of incurable neurodegenerative conditions that display a wide range of neurological symptoms including dementia, motor dysfunction, psychiatric problems and others [35]. The disease is caused by the replication, accumulation and spreading of misfolded and pathogenic prion proteins (PrP^SC^) in the nervous systems [36,37]. The main histopathological features are spongiform degeneration of the CNS, reactive gliosis, neuronal loss, chronic inflammation, and, in some cases, the formation of amyloid plaques. In ME7 scrapie-infected mice and Sporadic Creutzfeldt-Jakob disease (sCJD), the most common human prion disease, PAD2 and protein citrullination are significantly increased in reactive astrocytes in the brain. Several cytoskeletal, energy metabolism-associated, and myelin proteins were found to be citrullinated including vimentin, GFAP, enolase 1, aldolase A, MBP, cyclophilin A, and phosphoglycerate kinase [11,38,39].

MS is a chronic neurodegenerative disease characterized by inflammatory demyelination and axonal degeneration leading to a variety of neurological impairments to the visual, sensory, motor, cognitive and autonomic nervous systems [40]. These functional problems result from impaired neuronal function due to demyelination and axon degeneration. MS is generally thought to be an autoimmune disease mediated by T and B cell dysfunction leading to targeted destruction of myelin and axons [40,41]. The exact mechanism by which autoimmunity is triggered remains unknown. However, PAD2 and PAD4, and citrullinated proteins are elevated in myelin from MS patients and in active lesions with ongoing demyelination and surrounding reactive astrocytes [42,43]. Similarly, animal models of the disease show increased PAD2 and citrullinated MBP in the brain, particularly in the white matter [44,45]. Furthermore, MBP citrullination correlates to disease severity. In normal human brain, ~20% of MBP is citrullinated. In chronic MS, the citrullinated MBP increases to 45%. In the fulminating MS (Marburg disease), the citrullinated MBP accounted for 90% of the total [45,46].

## PAD2 and PC as Hallmarks of Reactive Astrogliosis in Neurodegenerative Diseases

An increase in PAD2 expression and PC in astrocytes is a common pathological phenomenon in neurodegenerative diseases. The colocalization of this increase with areas of neurodegeneration or injury suggests that the increase is an insult-induced response and may serve as markers of reactive astrogliosis [18]. What role PC plays in astrocytes and how PC contributes to neurodegeneration is unknown. Nonetheless, PC could impact astrocyte functions and exacerbate neurodegeneration. First, citrullination induces depolymerization and fragmentation of GFAP and other astrocytic intermediate filaments like vimentin [47-49]. This could interfere with the cytoskeletal architecture of the astrocytes, compromising its functions including structural and nutritional supports for neurons, and synaptic formations. Second, a byproduct of citrullination reaction is ammonia. Ammonia is implicated as a neurotoxicant in many diseases [50,51]. The overwhelming astrocytic protein citrullination may constitute an ammonia build-up that could impair the astrocyte and/or spill over to exacerbate neuronal damage.

## PC in Myelin Protein Aggregation and Myelin Degeneration

Protein aggregation is a prominent feature in neurodegenerative disease. Different protein aggregates not only differentiate distinct neurodegenerative diseases but also are thought to drive distinct neuropathology in each disease [52]. PTMs may modulate protein aggregation. For example, phosphorylation at specific sites of various proteins is enriched in disease-specific protein aggregates. In ALS and frontotemporal lobar degeneration (FTLD), phosphorylation of TDP-43 at S409 and S410 is enriched in TDP-43 aggregates [53,54]. Antibodies against the phosphorylation sites are widely used to detect the aggregates and as a neuropathological diagnostic marker. In PD and Lewy body dementia, phosphorylation of α-synuclein at S129 promotes aggregation, whereas phosphorylation at Y136 inhibits aggregation [55,56]. In AD and FTD-tau, hyperphosphorylated tau is enriched in tau aggregates and may facilitate and/or stabilize the aggregates and mediate neuronal toxicity [57].

Compared to phosphorylation, the relationship between PC and protein aggregation is much less studied and has only recently come to light. Yusuf and colleagues reported a progressive increase in citrullinated protein aggregates in white matter, starting at disease onset and peaking at the paralysis stage in ALS mouse models [18]. They also confirmed the existence of citrullinated protein aggregates in human ALS spinal cord and motor cortex [17]. The citrullinated aggregates colocalize with MBP and PLP aggregates, suggesting that PC may drive myelin protein aggregation. Previous literature suggests that myelin protein citrullination and aggregation may not be unique to ALS and may be relevant to other neurodegenerative diseases. In prion disease, an antibody raised against citrullinated MBP showed a punctate staining pattern in white matter [58], which could be MBP aggregates similar to what was observed in ALS. In an experimental autoimmune encephalomyelitis model (a model for MS), aggregate-like and PC-positive structures similar to those observed in ALS were detected in white matter, and increased levels of citrullinated MBP were detected in the insoluble fractions of brain and spinal cord proteins [59]. These results suggest that citrullinated protein aggregates that are enriched in myelin proteins may be common amongst various neurodegenerative conditions.

How PC impacts neurodegeneration is yet to be defined. In MS, citrullinated myelin proteins may represent autoantigens, thereby exacerbating autoimmunity against myelin and accelerate myelin destruction [3]. Moscarello and colleagues proposed that high levels of MBPcitrullination disrupt compact myelin by diminishing the electrostatic interactions between the positively charged arginines in MBP and the negatively charged phospholipids [60]. The citrullinated MBP becomes membrane-unbound, hasan open and unfolded structureand is vulnerable to proteolysis, which releases immunodominant peptides, triggering the immune response and destruction of myelin [61]. In ALS, increased MBP citrullination may result in its dissociation from the myelin membrane, misfolding, and subsequently aggregating. PC may also directly promote protein aggregation, as has been demonstrated with the myelin protein MOG, where citrullination promoted amyloid formation [62]. PC in myelin proteins may contribute to myelin degeneration in neurodegenerative diseases. However, PC may not just negatively impact neurodegeneration. Because numerous proteins are citrullinated and show complex changes in disease conditions, it may not be surprising that in some situations, citrullination in some proteins may be beneficial. Further experiments will be needed to tease out the significance of PC in neurodegenerative diseases.

## Citrullinated Proteins as Potential Biomarkers in ALS

Biomarkers are critical for ALS diagnosis and over the last decade, several biomarkers for ALS have been identified [63]. These include neuronal cytoskeleton specific proteins such as neurofilaments (NFs) that are released into the CSF and blood due to axon degeneration [64]. Currently, neurofilament light chain (NfL) is the leading biomarker for monitoring the clinical progression of ALS [65]. However, some reports have shown that phosphorylated NfH (pNfH) has more diagnostic sensitivity and specificity than NfL [66-68], suggesting that measuring protein PTMs may be advantageous for better diagnosing the disease. NfL and NfH as well as other neuronal skeletons, i.e. α-internexin and the microtubule-associated protein tau, are known to be citrullinated [28,69,70]. Furthermore, neuronal PC is decreased in ALS, and studies in ALS mouse models suggest that the decrease starts at disease onset [17,18]. The decreased PC may be an early signal for compromised neuronal function before neuronal loss, raising the possibility that measuring neuronal protein citrullination (e.g. citNfL, citNfH) from biofluids may improve the accuracy of current biomarkers for ALS diagnosis. There is also a possibility that citrullination of other proteins or peptides may be altered and could be biomarkers for ALS, even if their protein levels do not change in biofluids.

## Tools for Identifying Citrullinated Proteins

As changes in PC are being increasingly documented in neurodegenerative diseases, identifying citrullinated proteins and the changes in their citrullination sites has become increasingly important for understanding how citrullination modulates protein function and contributes to diseases. Several techniques have been developed in recent years to detect citrullinated proteins [8]. The easiest and quickest techniques are antibody-based. Senshu and colleagues developed an anti-citrulline modified (ACM) assay where a polyclonal antibody was raised against citrullinated proteins that were modified with 2,3-butanedione and antipyrine [71]. Nicholas and Whitaker developed another monoclonal antibody, F95, against a deca-citrullinated peptide [72]. Both antibodies recognize multiple citrullinated proteins and are widely used to detect citrullinated proteins in Western blots, immunohistochemistry, and immunofluorescence assays. These antibody assays detect the most abundant citrullinated proteins such as GFAP, vimentin, MBP and keratins when combined with other specific protein identification techniques, but they do not detect low-abundant citrullinated proteins nor the specific citrullination sites.

To identify citrullinated proteins efficiently, Lewallen and colleagues developed rhodamine- phenylglyoxal (RhPG) and biotin-PG (BPG) probes [73,74]. RhPG can be used in a high throughput assay format and can directly visualize protein citrullination in a complex biological proteome on an SDS-PAGE gel without transfer to a membrane. BPG can be used to enrich citrullinated proteins from cells or tissues, and the proteins can be visualized in western blots with streptavidin as a surrogate antibody. Furthermore, citrullinated proteins can be identified by liquid chromatography and tandem mass spectrometry (LC-MS/MS) [74]. By this approach, the whole citrullinome can be determined for both normal as well as diseased tissues [75].

A limitation of the BPG-enrichment coupled with LC-MS/MS method is that it does not pinpoint citrullinated amino acid residues in proteins. To solve this problem, several obstacles need to be overcome using standard mass spectrometry approaches. For example, it is difficult to identify deimination events, because the mass change is small (0.98 Da). Additionally, trypsin does not cleave after citrulline leading to longer peptides that are difficult to detect by MS/MS. These problems are further exaggerated when attempting to analyze low-abundance proteins in a complex biological sample. Lee and colleagues used LC-MS/MS to identify citrullination sites from various human tissues, but their work required manual inspection of mass spectra [76]. This approach is labor intensive, thereby limiting its wide use. Several researchers have tried to overcome this limitation by chemically modifying citrulline and detecting the mass shift of each adduct [77,78]. Recently, Maurais, Salinger and colleagues developed a software solution called ionFinder to automate this process [79]. Ionfinder was designed to unambiguously identify citrullinated peptides using a decision tree algorithm that takes into account the presence of neutral loss with the corresponding −43 Dalton shift on the mass spectra. The input data must be non-enriched proteomic data with protein coverage that spans the sites in question. Generally, it identifies highly abundant citrullinated proteins. There remains an opportunity to develop a MS spectrometry-based method that identifies the sites of citrullination on low abundant proteins.

## PAD2 as a Potential Therapeutic Target in ALS

Research has shown that motor neuron degeneration in ALS is triggered by multiple distinct and sometimes overlapping pathways. Therefore, treatments targeting upstream causes for all ALS including sporadic and familial cases may be difficult if not impossible. Consequently, targeting a downstream convergent pathway of this disease may have advantages as to the broad relevance. Our recent studies indicate that an increase in protein citrullination is a common pathway downstream of diverse neurodegenerative conditions, including ALS [17,18]. Preclinical studies targeting PAD2, the most abundant enzyme that catalyzes protein citrullination in the brain, has shown benefits in some disease models, thereby highlighting the therapeutic potential of PAD2 inhibition [12,80-84]. PAD inhibitors which are derivatives of benzoylated arginines include the pan-PAD inhibitors Cl-amidine and BB-Cl-amidine, and isoform-specific inhibitors AFM-30a and GSK199 for PAD2 and PAD4, respectively [85,86]. These compounds have shown efficacy in multiple inflammatory disease models [82,83,87-93]. Whether PAD2 inhibition is therapeutically beneficial in ALS and other neurodegenerative diseases remains to be investigated. Considering the similarities in PC between ALS and MS [17,18,42], and PAD2 targeting reduces citrullination and disease phenotypes in the latter [81], PAD inhibition may elicit similar beneficial outcomes in ALS. Further experiments will be needed to test this hypothesis.

## Conclusion

PC is a widespread PTM in various tissues and its alterations have been reported in the CNS of multiple neurodegenerative diseases. Detailed studies in ALS have demonstrated that PC is increased spatially and temporally in correlation with neurodegeneration. The upregulation of PC is mostly in astrocytes. By contrast, PC is decreased in neurons. Additionally, PC is enriched in myelin protein aggregates containing PLP and MBP. These observations, along with the observations in other neurodegenerative diseases, indicate that aberrant PC is a common hallmark in neurodegenerative diseases. To understand the significance of PC changes, future experiments will need to focus on the impact of these changes on the diseases. To pave the way for future experiments, the citrullinated proteins and the citrullination sites on these proteins need to be identified, and their changes in the diseases determined. To accomplish this, continuing efforts are needed to develop more powerful methods to efficiently unveil the citrullinome.

## Figures and Tables

**Table 1.** Overview of PADs and PC Changes in Neurodegenerative Diseases.

	Amyotrophic Lateral Sclerosis	Alzheimer’s Disease	Parkinson’s Disease	Multiple Sclerosis	Prion disease
Disease features	Progressive motor neuron loss; motor dysfunction and paralysis; aggregation of TDP-43 and other proteins [20].	Loss of cortical and hippocampal neurons; memory loss and dementia; accumulation of senile plaques and neurofibrillary tangles [23].	Progressive loss of dopaminergic neurons; motor dysfunction; neuronal α-synuclein inclusions [29].	Chronic inflammatory demyelination and axonal degeneration; functional impairment in sensory, motor, visual, and autonomic nervous systems [40].	Wildspread neurodegeneration in the CNS; accumulation and spreading of misfolded and pathogenic prion proteins in the nervous system [37].
CNS regions with altered PADs and/or PC	Motor cortex, Spinal cord and brainstem.	Hippocampus, frontal cortex, retina.	Substantia nigra, Prefrontal cortex, Cingulate cortex, and hippocampus.	Medulla, spinal cord, brain white matter.	Hippocampus, brain stem, striatum, thalamus, Frontal cortex.
PADs and PC associated cell type changes and pathology	↑ PAD2 and PC in reactive astrocytes in motor cortex and spinal cord [17,18]. ↓ PAD2 and PC in neurons [18]. ↑ PC in white matter aggregates [17,18]. PC enriched in myelin proteins MBP and PLP aggregates [17,18].	↑ PAD2 and PC in reactive astrocytes [24-26]. ↑ PAD4 and PC in cortical pyramidal and hippocampal hilar neurons [26]. ↑ PC in nucleus of neurons with cytoplasmic p-Tau [25]. PC colocalizes with Aβ42 in extracellular plaque [25]. ↑ citAβ in sporadic and familial AD [27]. ↑ PAD2, PAD4, citTau in AD models and postmortem brain and retinal tissues [70,94].	↑ PC in reactive astrocytes in substantia nigra in PD [16]. ↑ PC in dopamine neurons in substantia nigra in PD [16]. ↑ PAD2, PAD4, and citH3 in prefrontal cortex of X-linked Dystonia Parkinsonism (XDP) [32]. ↑ PADs and PC in anterior cingulate cortex and hippocampus in earlier PD stages [33]. ↑ PC in the vasculature of cortex and hippocampus in premotor PD rat model [34].	↑ PC in reactive astrocytes in areas of ongoing demyelination in chronic active lesions [42]. ↑ PC at plaque interfaces and gray-white matter junctions in MS brains [49]. ↑ PAD2, PAD4 and PC in MS brain normal-appearing white matter (NAWM) [14]. ↑ PAD activity and citMBP in MS mouse model [15]. ↑ PAD2, PAD4, and PC in myelin of MS brain [43]. ↑ PC stained as puncta in acute and relapse EAE mouse model in medulla and spinal cord [59].	↑ PAD2 and PC in reactive astrocytes in the brain of sCJD and ME7 scrapie-infected mice [11,38]. ↑ citVimentin accumulates in reactive astrocytes in sCJD and ME7 scrapie-infected mice [39]. ↑ citMBP stained as puncta in scrapie infected mice brain white matter [58].
Common modified proteins	PC overlaps with GFAP, and MBP and PLP aggregates [17,18].	Vimentin, GFAP, HSP90A, NFL, MBP, CNP [24,26,28].	NFM, NFH, HSP70, H2B, H4 [34]. PC overlaps with GFAP [16].	H3, MBP, GFAP, Vimentin, CN37, NFM [42,49,69].	Vimentin, GFAP, MBP [11].
Models studied	Mouse models, SOD1G93A and PFN1C7G. Sporadic ALS postmortem tissues.	AD postmortem brain and retinal tissues. AD tauopathy mouse model.	PD postmortem brain tissues. XDP postmortem tissues. Rat model of pre-motor PD.	DM-20 overexpressing mice, PAD2 overexpressing mice, EAE mouse model, MS postmortem tissues.	sCJD postmortem brain and ME7 scrapie-infected mice.
